# Serious Gaming and Gamification Education in Health Professions: Systematic Review

**DOI:** 10.2196/12994

**Published:** 2019-03-28

**Authors:** Sarah Victoria Gentry, Andrea Gauthier, Beatrice L’Estrade Ehrstrom, David Wortley, Anneliese Lilienthal, Lorainne Tudor Car, Shoko Dauwels-Okutsu, Charoula K Nikolaou, Nabil Zary, James Campbell, Josip Car

**Affiliations:** 1 Norwich Medical School University of East Anglia Norwich United Kingdom; 2 Department of Primary Care and Public Health School of Public Health Imperial College London London United Kingdom; 3 Institute of Medical Science Faculty of Medicine University of Toronto Toronto, ON Canada; 4 Department of Learning, Informatics, Management and Ethics Karolinska Institutet Stockholm Sweden; 5 Royal Society of Arts London United Kingdom; 6 Family Medicine and Primary Care Lee Kong Chian School of Medicine Nanyang Technological University Singapore Singapore; 7 Centre for Population Health Sciences Lee Kong Chian School of Medicine Nanyang Technological University Singapore Singapore; 8 Centre de Philosophie du Droit (Cellule Biogov) Universite Catholique de Louvain Louvain-la-Neuve Belgium; 9 Games for Health Innovations Centre Lee Kong Chian School of Medicine Nanyang Technological University Singapore Singapore; 10 Emerging Technologies Lab Mohammed VI University of Health Sciences Casablanca Morocco; 11 Health Workforce Department World Health Organization Geneva Switzerland

**Keywords:** video games, education, professional, review

## Abstract

**Background:**

There is a worldwide shortage of health workers, and this issue requires innovative education solutions. Serious gaming and gamification education have the potential to provide a quality, cost-effective, novel approach that is flexible, portable, and enjoyable and allow interaction with tutors and peers.

**Objective:**

The aim of this systematic review was to evaluate the effectiveness of serious gaming/gamification for health professions education compared with traditional learning, other types of digital education, or other serious gaming/gamification interventions in terms of patient outcomes, knowledge, skills, professional attitudes, and satisfaction (primary outcomes) as well as economic outcomes of education and adverse events (secondary outcomes).

**Methods:**

A comprehensive search of MEDLINE, EMBASE, Web of Knowledge, Educational Resources Information Centre, Cochrane Central Register of Controlled Trials, PsycINFO, and Cumulative Index to Nursing and Allied Health Literature was conducted from 1990 to August 2017. Randomized controlled trials (RCTs) and cluster RCTs were eligible for inclusion. Two reviewers independently searched, screened, and assessed the study quality and extracted data. A meta-analysis was not deemed appropriate due to the heterogeneity of populations, interventions, comparisons, and outcomes. Therefore, a narrative synthesis is presented.

**Results:**

A total of 27 RCTs and 3 cluster RCTs with 3634 participants were included. Two studies evaluated gamification interventions, and the remaining evaluated serious gaming interventions. One study reported a small statistically significant difference between serious gaming and digital education of primary care physicians in the time to control blood pressure in a subgroup of their patients already taking antihypertensive medications. There was evidence of a moderate-to-large magnitude of effect from five studies evaluating individually delivered interventions for objectively measured knowledge compared with traditional learning. There was also evidence of a small-to-large magnitude of effect from 10 studies for improved skills compared with traditional learning. Two and four studies suggested equivalence between interventions and controls for knowledge and skills, respectively. Evidence suggested that serious gaming was at least as effective as other digital education modalities for these outcomes. There was insufficient evidence to conclude whether one type of serious gaming/gamification intervention is more effective than any other. There was limited evidence for the effects of serious gaming/gamification on professional attitudes. Serious gaming/gamification may improve satisfaction, but the evidence was limited. Evidence was of low or very low quality for all outcomes. Quality of evidence was downgraded due to the imprecision, inconsistency, and limitations of the study.

**Conclusions:**

Serious gaming/gamification appears to be at least as effective as controls, and in many studies, more effective for improving knowledge, skills, and satisfaction. However, the available evidence is mostly of low quality and calls for further rigorous, theory-driven research.

## Introduction

Innovative approaches and modalities for education in health professions education are constantly sought to improve teaching and learning and ultimately patient care and outcomes. Digital education may be one such innovation. This review focuses on serious gaming and gamification education.

For the purposes of this review, we have used the terminology defined by Alvarez [1,2]. The term “serious game” was used to refer only to games designed specifically for the “serious” purpose of providing health professions education via a digital device. The term “serious diverting” was used to refer to the use of games originally designed primarily for entertainment used without modification, as part of health professions education delivered via a digital device. “Serious gaming” was used to refer to any use of digital games for health professions education, thereby encompassing “serious games” and “serious diverting.”

A related but separate concept—“gamification”—can be defined as “the application of the characteristics and benefits of games to real world processes or problems” [3]. Gamification differs from serious games in terms of the design intention, with gamification interventions involving the application of game elements with a utilitarian purpose and serious games designed as full-fledged games for a purpose other than just entertainment [4]. Wortley suggests that both may be experienced by the user as a complete game, although typically, gamification involves the use of game components outside a game setting, such as rewarding users completing an electronic learning (e-learning) module with badges or points. Gamification has the potential to allow for greater involvement of the user in setting his/her own objectives or outcomes, personalization of the intervention, and cost-effectiveness [3]. Most, but not all, uses of the term refer to interventions involving the use of enabling digital technologies.

Serious gaming/gamification has the potential to provide learners with opportunities to be part of active learning, solving clinical problems, and gaining experience in risk-free surroundings [5], without needing to involve patients. Learners may have the opportunity to develop analytical skills, strategic thinking, knowledge, multitasking, decision making, communication, and psychomotor skills [6], with multiplayer functions providing opportunities for collaborative learning [7]. The motivational properties of gaming have the potential to be harnessed for educational purposes [8]. Serious gaming/gamification can be used at a time and place that suits the learner. The reusable nature of serious gaming/gamification may allow more frequent or longer interactions, free up lecturer time, and provide monetary savings [9]. However, this could lead to reduced opportunities to ask questions, hold discussions, and spend time with patients. Use of such interventions within small groups, with lecturer support, could allow for discussion and interaction but would likely increase lecturer time needed as compared to traditional learning. Serious gaming/gamification, like other kinds of e-learning, may ease the process of updating materials, as modifications to content can be made continuously, unlike with a text book.

Although serious gaming and gamification interventions appear to have much potential, rigorous evaluation is required to assess whether they can lead to effective learning. There is a potential for the game or game elements to become a distraction rather than a facilitator of learning, with the method “more memorable than the message” [10]; therefore, the quality of learning must be the focus, as opposed to the capabilities of the technology used [11].

This systematic review is one of a series of reviews evaluating the scope for implementation and potential impact of a wide range of digital health education interventions for pre- and postregistration health professions. This review was conducted in collaboration with the World Health Organization’s Health Workforce Department. The objective of this work is to compare the effectiveness of serious gaming and gamification education versus various controls in improving learners’ knowledge, skills, professional attitudes, and satisfaction as well as patient outcomes.

## Methods

While conducting and reporting the review, we adhered to the gold-standard systematic review methods recommended by the Cochrane Collaboration [12]. For a detailed description of the methodology, please refer to our previous paper [13].

### Search Strategy and Data Sources

We comprehensively searched the following databases between 1990 and August 2017: MEDLINE (Ovid), EMBASE (Elsevier), Web of Science, Educational Resource Information Centre (Ovid), Cochrane Central Register of Controlled Trials (CENTRAL), (The Cochrane Library), PsycINFO (Ovid), and Cumulative Index to Nursing and Allied Health Literature (EBSCO). The search strategy for MEDLINE is presented in the Multimedia Appendix 1. We searched for papers in English but considered eligible studies in any language. We also searched two trials registries, reference lists of all included studies, and relevant systematic reviews and contacted the relevant investigators for further information.

### Eligibility Criteria

We included randomized controlled trials (RCTs) and cluster RCTs (cRCTs) of pre- and postregistration health professions using serious gaming/gamification with any type of controls (traditional learning, digital education, or another type of serious gaming/gamification intervention), which measured patient outcomes, knowledge, skills (cognitive and psychomotor), professional attitudes, and satisfaction (primary outcomes) and adverse effects or costs (secondary outcomes). We excluded crossover trials due to the high likelihood of carry-over effect. Participants were not excluded on the basis of sociodemographic characteristics such as age, gender, ethnicity, or any other related characteristics. Outcome definitions are available in the associated paper [13].

### Data Selection, Extraction, and Management

The search results from different electronic databases were combined in a single EndNote library (X 8.2; Clarivate Analytics, Philadelphia, PA), and duplicate records were removed. Two reviewers independently screened titles and abstracts to identify studies that potentially met the inclusion criteria. The full texts of these articles were retrieved and read. Two review authors independently assessed these articles against the eligibility criteria (SG, AG, and BE). At least two reviewers independently extracted the data for each of the included studies using a structured data-extraction form. We extracted all relevant data on the characteristics of participants, interventions, controls, and outcomes measures. For continuous data, we reported standardized mean differences and SDs. None of the studies reported dichotomous data. Disagreements were resolved through discussion.

### Assessment of Risk of Bias

Two reviewers independently assessed the risk of bias of the included studies using the Cochrane Collaboration’s “Risk of bias” tool [12]. Studies were assessed for the risk of bias in the following domains: random sequence generation, allocation concealment, blinding (participants and personnel), blinding (outcome assessment), completeness of outcome data (attrition bias), selective outcome reporting (reporting bias), and other risk of bias. For cRCTs, we also assessed recruitment bias, baseline imbalances, loss of clusters, and incorrect analysis. Judgements concerning the risk of bias for each study were classified as high, low, or unclear.

### Data Synthesis

Data were synthesized using Review Manager (Version 5.3; The Nordic Cochrane Centre, The Cochrane Collaboration, Copenhagen, Denmark). Included studies were insufficiently homogenous in terms of population, inclusion criteria, interventions, and outcomes for meta-analysis. The decision not to perform a meta-analysis was made by a consensus of review authors. We present a narrative synthesis of findings, with effect sizes calculated for outcomes where there were sufficient data. Where possible, we assessed the quality of studies and size of effect. Results are presented by outcome and separately for each comparison (serious gaming/gamification vs traditional learning, serious gaming/gamification vs digital health education, and serious gaming/gamification vs serious gaming/gamification).

### Assessment of Evidence Quality

The results for comparisons between serious gaming/gamification and traditional learning as well as serious gaming/gamification and digital education are presented in the narrative summary of findings tables (Tables 7 and 8). Two authors (SG and AG) rated the overall quality of the evidence as implemented and described in GRADEprofiler (GRADEproGDT online version; Evidence Prime, Inc, Hamilton, ON, Canada) and chapter 11 of the Cochrane Handbook for Systematic Reviews of Interventions [12]. We considered the following criteria to assess the quality of the evidence: limitations of studies (risk of bias), inconsistency of results, indirectness of the evidence, imprecision, and publication bias. We also downgraded the quality, where appropriate. This was done for all primary outcomes reported in the review.

## Results

**Figure 1:**
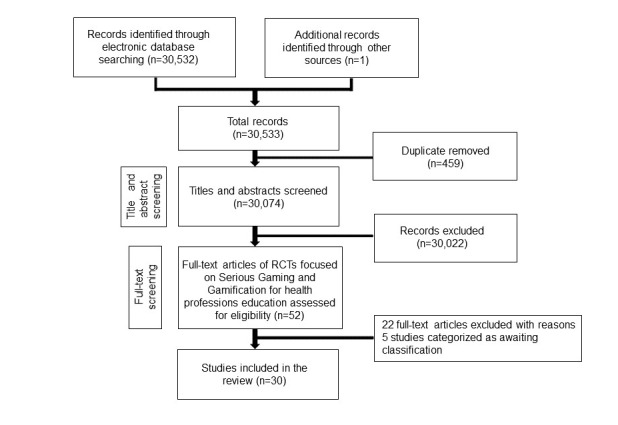
PRISMA flow chart. PRISMA: Preferred Reporting Items for Systematic Reviews and Meta-Analyses; RCT: randomized controlled trial.

Our searches yielded a total of 30,532 citations and 30 studies (27 RCTs and 3 cRCTs) including 3634 participants (Figure 1).

### Included Studies

#### Study Designs and Populations

Sample sizes ranged from 14 [14] to 1470 [15] participants. Almost half the included studies had sample sizes below 50.

Fourteen studies were conducted in Europe [16-30], and 11 studies were conducted in North America [14,15,31-40], one of which recruited participants from 63 countries via the internet [15]. One study was conducted in Singapore [41]. Four studies were conducted in middle-income countries, three of which were conducted in Brazil [42-44]. One study was conducted by authors based in China and Taiwan, but it was unclear where the study itself was carried out [45]. None of the included studies were conducted in low-income countries. Details of study designs and population for each trial are compared in Tables 1-3 and a summary is given below.

Eleven studies included only medical students [14,16,17,19,20,24-26,39,40,42,45]. Five studies included only nursing students [22,27,29,37,41] and four included only surgical residents [18,30,31,38]. The remaining studies included primary care doctors (n=2) [36,43]; dental students (n=2) [21,32]; anesthesiology residents (n=1) [35]; urologists (n=1) [15]; speech and language science students (n=1) [44]; participants of the Major Incident Medical Management and Support course, which typically includes doctors, nurses, and paramedics with an interest in prehospital care (n=1) [23]; nursing and medical students (n=1) [28]; and medical students, residents, and specialists in Obstetrics and Gynecology (n=1, reported in one article and one conference abstract) [33,34].

**Table 1:** Study designs and populations of the included studies comparing serious gaming/gamification and traditional learning.

Study	Study type	Population (n)	Country	Field of study
Adams et al 2012 [31]	RCT^a^	Surgical residents (31)	United States	General surgery
Boada et al 2015 [27]	RCT	Nursing students (109)	Spain	Cardiopulmonary resuscitation skills
Boeker et al 2013 [20]	RCT	Medical students (145)	Germany	Urology
Cook 2012 et al [22]	RCT	Nursing students (34)	United Kingdom	Intermediate life support
De Araujo et al 2016 [42]	RCT	Medical students (20)	Brazil	Surgical skills
Del Blanco et al 2017 [28]	RCT	Nursing and medical students (132)	Spain	Preparation for going into the operating theatre
Diehl et al 2017 [43]	RCT	Primary care physician (134)	Brazil	Insulin management in primary care

Foss et al 2014 [29]	RCT	Nursing students (201)	Norway	Medication calculation
Giannotti et al 2013 [30]	RCT	Surgical residents (42)	Italy	Surgical skills
Graafland et al 2017 [18]	RCT	Surgical residents (31)	Netherlands	Minimally invasive surgery
Hannig et al 2013 [21]	RCT	Dental students (55)	Germany	Alginate mixing skills
Katz et al 2017 [35]	RCT	Anesthesiology residents (44)	United States	Liver transplant anesthesiology

Knight et al 2010 [23]	cRCT^b^	Health professionals on a Major Incident Management Course (91, 2 clusters)	United Kingdom	Major incident management and support
Lagro et al 2014 [19]	cRCT	Medical students (145, 5 clusters)	Netherlands	Geriatrics
LeFlore et al 2012 [37]	RCT	Nursing students (106)	United States	Pediatric respiratory disease

Li et al 2015 [45]	RCT	Medical students (97)	China/Taiwan	Cardiopulmonary resuscitation
Plerhoples et al 2011 [38]	RCT	Surgical residents (40)	United States	Surgical skills
Rondon et al 2013 [44]	RCT	Speech-language and hearing science students (29)	Brazil	Anatomy and physiology
Tan et al 2016 [41]	cRCT	Nursing students (103, 7 clusters)	Singapore	Blood transfusion administration

^a^RCT: randomized controlled trial.

^b^cRCT: cluster randomized controlled trial.

**Table 2:** Study designs and populations of the included studies comparing serious gaming/gamification and other digital education interventions.

Study	Study type	Population (n)	Country	Field of study
Amer et al 2011 [32]	RCT^a^	Dental students (80)	United States	Operative dentistry
Chien et al 2013 [14]	RCT	Medical students (14)	United States	Laparoscopic surgical tasks
Dankbaar et al 2016 [16]	RCT	Medical students (79)	Netherlands	Approach to acutely unwell patients
Dankbaar et al 2017 [17]	RCT	Medical students (66)	Netherlands	Patient safety and stress management
Gauthier et al 2015 [40]	RCT	Medical students (44)	Canada	Vascular anatomy
Kerfoot et al 2014 [36]	RCT	Primary care physician (111)	United States	Management of blood pressure in primary care
Sward et al 2008 [39]	RCT	Medical students (100)	United States	Pediatrics


^a^RCT: randomized controlled trial.

**Table 3:** Study designs and populations of included studies comparing serious gaming/gamification and another type of serious gaming/gamification intervention.

Study	Study type	Population	Country	Field of study
Adams et al 2012 [31]	RCT^a^	Surgical residents (31)	United States	General surgery
De Araujo et al 2016 [42]	RCT	Medical students (20)	Brazil	Surgical skills
Hedman et al 2013 [24] and Kolga et al	RCT	Medical students (30)	Sweden	Surgical skills
Ju et al 2011 [34] and Ju et al 2012 [33]	RCT	Medical students, residents and attendings (42)	United States	Surgical skills

Kerfoot et al 2012 [15]	RCT	Urologists (1470)	United States (participants recruited online from 63 countries)	Urology guideline knowledge
Kolga et al 2008 [26]	RCT	Medical students (22)	Sweden	Surgical skills


^a^RCT: randomized controlled trial.

#### Interventions

Characteristics of the interventions included are compared in Tables 4-6 and a summary is given below.

Two of the included studies evaluated “gamification” interventions [15,36]. The remainder evaluated serious gaming interventions. Two of these were group interventions, in which a serious gaming intervention was projected to a traditional classroom of learners who played together [39,44].

A total of 22 interventions had original design purposes other than entertainment, of which 11 interventions were classified as “Message Broadcasting - Educative” [15,17,20,28,36,37, 39-41,43,44]; four were classified as “Training - Mental” [19,22,23,29], three were classified as “Training - Physical” [18,21,32], and four were classified as both “Training - Mental” and “Training - Physical” [16,27,35,45]. All of the interventions with design purposes other than entertainment were classified under “Education” for “Scope.”

The remaining eight interventions were commercial off-the-shelf games designed only for the purpose of entertainment but used for training of motor skills as part of “Serious Diverting” interventions. These were all classified as “Training - Physical” for “Purpose” and as “Entertainment” and “General Public” for “Scope” [14,24-26,30,31,33,34,38,42].

**Table 4:** Characteristics of included interventions in studies comparing serious gaming/gamification and traditional learning.

Study	Intervention type	Intervention duration	Intervention frequency	Intervention intensity	Control
Adams et al 2012 [31]	First-person shooter, commercial-off the-shelf intervention	6 weeks	Weekly	Mean of 5.7 (SD 1.3) hours	Box trainer
Boada et al 2015 [27]	Life support–simulation activities	Access for 1 week	—^a^	All did >50% of the tasks	Usual education
Boeker et al 2013 [20]	Electronic adventure game “Uro-Island”	Access for 1 week	—	—	Written script
Cook et al 2012 [22]	Platform for undergraduate life support education game	2 weeks	—	Unlimited access	Usual learning
De Araujo et al 2016 [42]	Surgical commercial-off-the shelf intervention (SurgG)	Access for 3 weeks	—	Mean of 647 minutes per week	Usual learning (ContG)
Del Blanco et al 2017 [28]	Videogaming intervention	Access for 1 day	Once	Variable	Usual learning
Diehl et al 2017 [43]	“InsuOnline” game	Access for 21 days	—	Mean of 4 hours	Onsite learning activity

Foss et al 2014 [29]	“The Medication Game” online training	Access for 4.5 weeks	—	—	Standard education
Giannotti et al 2013 [30]	Nintendo Wii training	4 weeks	5 days per week	60 minutes	Usual training
Graafland et al 2017 [18]	Game enhanced curriculum (Dr Game, Surgeon Trouble)	—	Two sessions	30 minutes	Usual training
Hannig et al 2013 [21]	Skills-O-Mat interactive game	60 minutes	Once	—	Teacher-catered workshop
Katz et al 2017 [35]	“OCT trainer” game where players work through the steps in liver transplant anesthesiology	30 days	81% self- reported playing 1-3 times per week	—	Usual training

Knight et al 2010 [23]	“Triage Trainer” computer game	60 minutes	Once	—	Card-sorting exercise
Lagro et al 2014 [19]	Geriatrics game in which players must balance patient-oriented goals and preferences, appropriateness of medical care, and costs	60-90 minutes	Once	—	Standard educational activity


LeFlore et al 2012 [37]	“Virtual Patient Trainer” game	2-3 hours	Once	—	Traditional lecture

Li et al 2015 [45]	3D cardiopulmonary resuscitation game	3 months (with 2-week extension possible)	—	—	Reminders to refresh their skills sent frequently
Plerhoples et al 2011 [38]	Commercial off-the-shelf intervention	10 minutes	Once	—	Standard educational activity
Rondon et al 2013 [44]	Computer game-based learning played as a group on a projector	9 weeks	Once per week	1 hour	Short scientific texts
Tan et al 2016 [41]	Videogame simulating blood transfusion–administration challenges and minigames	30 minutes	Once	—	Usual education



^a^Not available.

**Table 5:** Characteristics of included interventions in studies comparing serious gaming/gamification and other digital education interventions.

Study	Intervention type	Intervention duration	Intervention frequency	Intervention intensity	Control
Amer et al 2011 [32]	Interactive dental videogame	Up to 20 minutes	Once	—^a^	3-minute video on resin bonding
Chien et al 2013 [14]	3D tennis game	40 minutes	Once	—	Virtual simulator training platform
Dankbaar et al 2016 [16]	Computer-based simulation game “abcdeSIM”	Access for 4 weeks	—	Estimated to take 2-4 hours to complete; mean logged game time 90 (SD 49) minutes	Electronic module
Dankbaar et al 2017 [17]	“Air-Medic Sky-1” game	1 week	—	3-4 hours	Digital education module
Gauthier et al 2015 [40]	“Vascular Invaders” game	Access for 35 days	—	—	Vascular anatomy study aid (online)
Kerfoot et al 2014 [36]	Online spaced-education game (question emailed every 3 days; resent 12 or 24 days later if answered incorrectly or correctly, respectively; retired after answered correctly on >two consecutive attempts)	Access for 52 weeks	—	Mean of 38 (SD 7) weeks to complete the cycle of questions	Online posting
Sward et al 2008 [39]	Web-based pediatric board game	4 weeks	One per week	1 hour	Self-study Web flash cards

^a^Not available.

**Table 6:** Characteristics of included interventions in studies comparing serious gaming/gamification and another type of serious gaming/gamification intervention.

Study	Intervention type	Intervention duration	Intervention frequency	Intervention intensity	Control
Adams et al 2012 [31]	FPS^a^ COTS^b^ intervention	6 weeks	Weekly	Mean of 5.7 (SD 1.3) hours	Non-FPS COTS intervention
De Araujo et al 2016 [42]	Surgical COTS intervention (SurgG)	Access for 3 weeks	—^c^	Mean of 647 minutes per week	Usual learning (ContG), FPS COTS (ShotG), Racing COTS (RaceG) interventions
Hedman et al 2013 [24] and Kolga et al 2009 [25]	Systematic video game training with FPS COTS intervention	5 weeks	5 days per week	30-60 minutes	Non-FPS COTS intervention
Ju 2011 et al [34]) and Ju et al 2012 [33]	Wii COTS intervention	30 minutes	Once	—	Play Station 2 COTS intervention
Kerfoot et al 2012 [15]	Online spaced-education game - 4 questions every 4 days	8-42 days	—	—	Spaced-education game – 2 questions every 2 days
Kolga et al 2008 [26]	FPS COTS intervention	5 weeks	5 days per week	30 minutes	2D non-FPS COTS intervention

^a^FPS: first-person shooter.

^b^COTS: commercial off the shelf.

^c^Not available.

**Figure 2:**
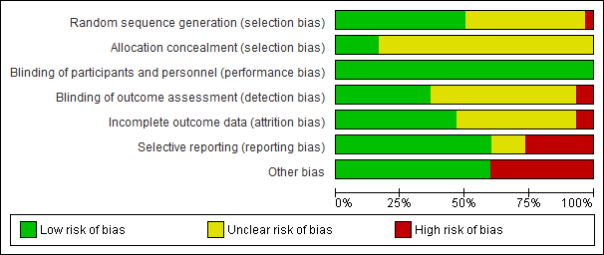
Risk-of-bias graph.

#### Comparisons and Outcomes

Serious gaming/gamification was compared with traditional learning in 19 studies [18-23,27-31,35,37,38,41-45], with digital education in 7 studies [14,16,17,32,36,39,40], and with other serious gaming/gamification interventions in 6 studies [15,24-26,31,33,42].

One study addressed patient outcomes [36]. Fourteen studies assessed knowledge [15,17,19,20,28,32,36,37,39-41,43,44]. Twenty-three studies addressed outcomes relating to skills [14,16-19,21-35,37-39,41,42,45]. Four studies assessed outcomes related to attitudes [17,24,25,28,43]. Sixteen studies addressed participant satisfaction [15-17,19-22,26,27,29,32, 37,39,42,43,45].

#### Risk of Bias in Included Studies

**Figure 3:**
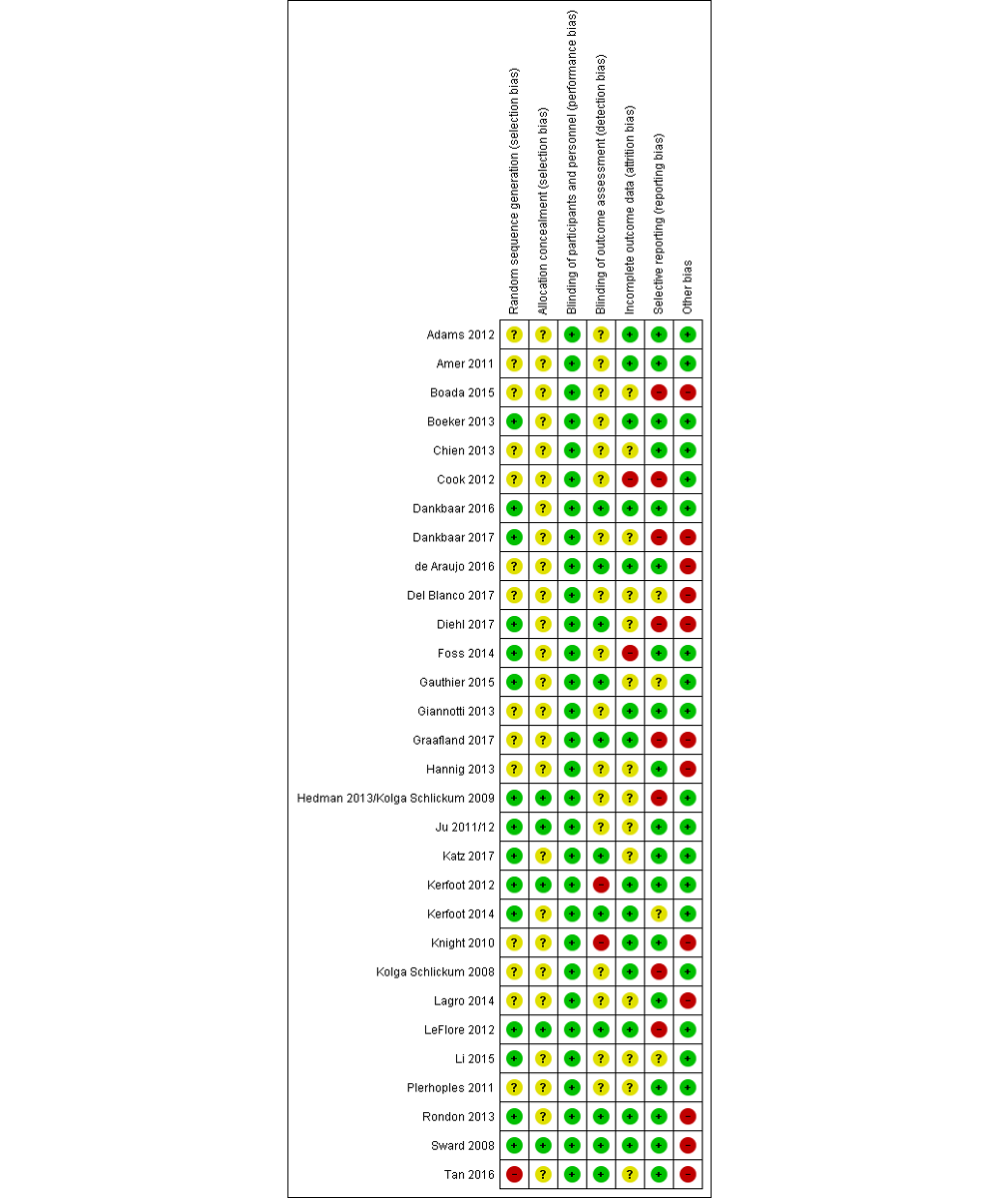
Risk-of-bias summary.

Figures 2 and 3 summarize the risk-of-bias assessments for the included studies. A total of 25 of the included studies were considered to be at high risk of bias [14,16-23,26-32, 35,36,38,40-45] according to Cochrane guidelines, because they had a high or unclear risk of bias for either the sequence generation or allocation concealment domains [12]. All three cRCTs were rated high for incorrect analysis, as none accounted for clustering in the analysis.

#### Effects of Interventions

Effects of the interventions are compared in Multimedia Appendices 2-4 and a summary is given below.

**Figure 4:**
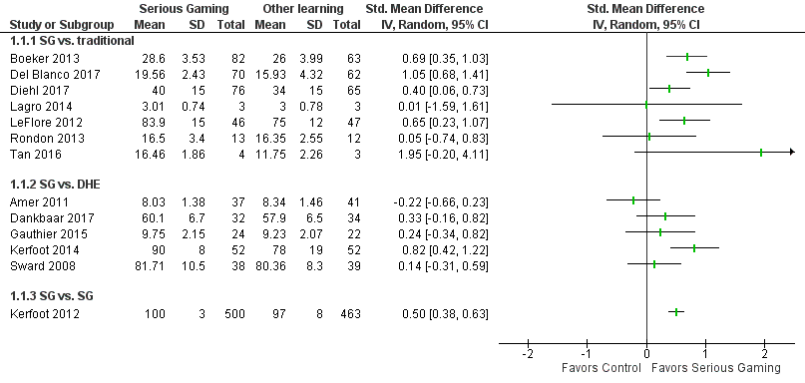
Forest plot for knowledge outcomes. IV: inverse variance; SG: serious games; DHE: digital health education.

### Primary Outcomes

#### Patient-Related Outcomes

One study measured patient-related outcomes [36]. This study compared serious gaming/gamification with an online posting intervention for primary care physicians and reported significantly shorter time to control blood pressure in the intervention group for only a subgroup of participants whose patients were already on antihypertensive medication at the start of the study (*P*=.02), although this may not be clinically significant (117 vs 125 days). Data were insufficient for calculation of standardized mean differences (SMD). The quality rating assessed using Grading of Recommendations, Assessment, Development and Evaluations (GRADE) was low.

#### Knowledge

Figure 4 summarizes the results of studies reporting knowledge outcomes.

#### Serious Gaming/Gamification Versus Traditional Learning

Four RCTs reported higher posttest scores in a serious gaming/gamification group than in a traditional learning group, with a mostly moderate magnitude of effect [20,28,37,43]. Interventions included a videogame to prepare students to enter the operating room [28] (SMD 1.05, 95% CI: 0.68-1.41), a urology educational adventure game [20] (SMD: 0.69, 95% CI: 0.35-1.03), pediatric respiratory disease-assessment game for nurses [37] (SMD: 0.65, 95% CI 0.23-1.07), and the InsuOnline serious game [43] (SMD: 0.40, 95% CI: 0.06-0.73). Comparisons were made between usual learning, written script, and traditional lectures.

One RCT of a group intervention, where speech and language science students played a serious game together in a classroom via a projector, found no difference in anatomy and physiology knowledge compared to a self-study control [44] (SMD: 0.05, 95% CI: –0.74 to 0.83).

Two cRCTs were also included [19,41]. One showed evidence of a large magnitude of effect for a blood transfusion serious game as compared to usual education, although the effect may not have been statistically significant (SMD: 1.95, 95% CI: –0.20 to 4.11) [41]. The second study showed no evidence of effect for a geriatric game compared with standard education, although this study measured perceived knowledge rather than an objective measure (SMD: 0.01, 95% CI: –1.50 to 1.61) [19].

All the individually played games with an objective assessment of knowledge suggested that serious gaming/gamification was superior to traditional learning. The quality rating assessed using GRADE was low for this outcome and comparison (Table 7).

#### Serious Gaming/Gamification Versus Other Modalities of Digital Education

Five studies found no evidence of a difference. Studies included comparison of serious gaming on dentin bonding and an online lecture control [32] and serious gaming with digital education on patient safety [17] and Web-based vascular anatomy study aids with and without game elements [40]. One study of a group serious gaming intervention found no difference in pediatric knowledge between groups who played a projected board game in teams in a conference room with Web-based pediatric flashcards [39]. One study found that compared with an online educational posting, serious gaming (an online spaced-education game) may improve knowledge (large magnitude of effect) [36] (SMD: 0.82, 95% CI: 0.42-1.22). The quality rating assessed using GRADE was low (Table 8).

**Table 7:** Summary of findings for serious gaming versus traditional learning. Patient or population: various health professionals, settings: high- and middle-income countries, intervention: serious gaming and gamification, comparison: traditional learning.

Outcomes	Number of participants (number of studies)	Quality of evidence (GRADE^a^)	Comments
Knowledge (measures include multiple-choice questions, clinical scenario–based questions, and self-assessment; follow-up mostly immediately after the intervention, longest follow-up of 52 weeks)	769 (7)	Low^b,c,d^	All the individually played games with an objective assessment of knowledge suggested serious gaming/gamification was superior to traditional learning. Four RCTs^e^ and one cRCT^f^ reported higher postintervention knowledge scores between the serious gaming and control groups, with moderate-to-large effect sizes, although the result for the cRCT may not have been statistically significant^g^. An RCT of a serious gaming intervention reported no difference between groups. A cRCT assessing perceived knowledge reported no difference between groups.
Skills (measures include performance metrics on a simulator, practical examinations, OSCEs^h^ and self-evaluation; most studies followed up until immediately after the intervention only)	1195 (14)	Low	Six RCTs reported higher postintervention skill scores on all measures of skills employed in that study in the serious gaming group, with small-to-large effect sizes. A further cRCT suggested higher skill scores of small magnitude but may not have been statistically significant^g^. Three RCTs measured skill outcomes using multiple measures (and no summary measure) and reported higher postintervention scores for some of these measures and no difference for others. Two RCTs and one cRCT reported no difference in postintervention skill scores between groups. One cRCT suggested serious gaming may be inferior to traditional learning, but this result may not have been statistically significant^g^.
Attitudes (measured with participant-completed rating scales; follow-up immediately after the test)	369 (3)	Very low^b,c,i,^^j^	One RCT reported higher postintervention attitude scores in the serious gaming group (small effect size) and one RCT reported no difference between groups. One reported higher scores in the intervention groups, but this result may not have been statistically significant^g^.
Satisfaction (3 questions on attitudes toward learning experience measured on a 4-point Likert scale; follow-up immediately after the intervention)	144 (1)	Low	One study reported higher postintervention satisfaction scores in the serious gaming group compared with the control.

^a^GRADE: Grading of Recommendations, Assessment, Development and Evaluations.

^b^Rated down one level for study limitations: The risk of bias was unclear for multiple domains.

^c^Rated down one level for imprecision: All included studies assessing this comparison and outcome had fewer than 400 participants.

^d^Low quality (+ + – –): Further research is very likely to have an important impact on our confidence in the estimate of effect and is likely to change the estimate.

^e^RCT: randomized controlled trial.

^f^cRCT: cluster randomized controlled trial.

^g^None of the 3 included cRCTs accounted for clustering in their analyses. They were therefore reanalyzed using the number of clusters as the sample sizes and were likely significantly underpowered.

^h^OSCE: objective structured clinical examination.

^i^Rated down one level for inconsistency: There was considerable heterogeneity in the results without a clear explanation.

^j^Very low quality (+ – – –): We are uncertain about the estimate.

**Table 8:** Summary of findings for serious gaming versus other modalities of digital education. Patient or population: health professionals in education, settings: high-income countries, intervention: serious gaming and gamification, comparison: other modalities of digital education.

Outcomes	Number of participants (number of studies)	Quality of evidence (GRADE^a^)	Comments
Patient outcomes (blood pressure)	111 (1)	Low^b,c,d^	One study reported better scores for blood pressure in some subgroups. Effect sizes could not be estimated due to missing data.
Knowledge (measures include multiple-choice questions and clinical scenario–based questions; follow-up mostly immediately after the intervention)	403 (5)	Low	One study reported higher scores in the serious gaming group with a large magnitude of effect. Four studies reported no difference.
Skills (measures include performance metrics on a simulator, practical examinations, OSCEs^e^, and self-evaluation; most studies followed up until immediately after the intervention only)	290 (5)	Low	One study reported superior scores in the virtual reality control group compared with the serious gaming intervention group. Two studies reported no difference. Two studies reported insufficient data for calculation of effect sizes.
Attitudes (measured with participant-completed rating scales; follow-up immediately after the test)	66 (1)	Low	One study reported no difference in postintervention attitudes scores between groups.
Satisfaction (measured with participant-completed rating scales; follow-up immediately after the test)	245 (3)	Low	Three studies reported higher satisfaction scores in the serious gaming group than groups of other modalities of digital education.

^a^GRADE: Grading of Recommendations, Assessment, Development, and Evaluations.

^b^Rated down one level for imprecision: All included studies assessing this comparison and outcome had fewer than 400 participants.

^c^Rated down one level for inconsistency: There was considerable heterogeneity in the results without a clear explanation.

^d^Low quality: Further research is very likely to have an important impact on our confidence in the estimate of effect and is likely to change the estimate.

^e^OSCE: objective structured clinical examination.

**Figure 5:**
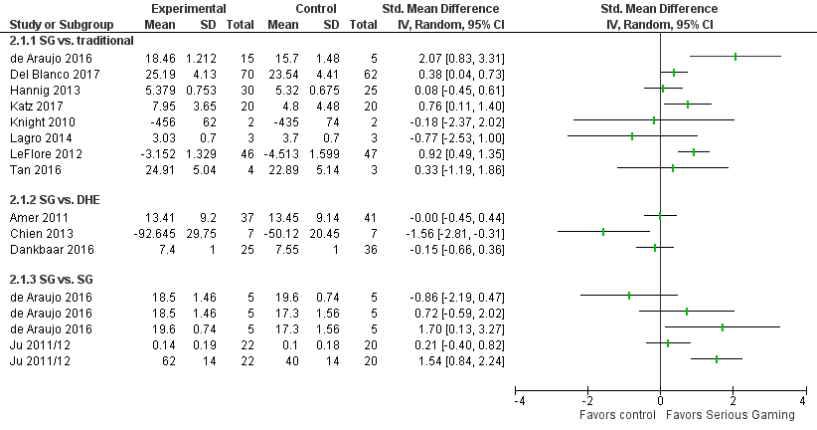
Forest plot for skills outcomes. IV: inverse variance. SG: serious games; DHE: digital health education.

#### Serious Gaming/Gamification Versus Serious Gaming/Gamification

One study of a spaced-education game found that interventions with greater question spacing (four questions every 4 days rather than two questions every 2 days) resulted in higher posttest scores, with a moderate magnitude of effect [15] (SMD: 0.50, 95% CI: 0.38-0.64). The quality rating assessed using GRADE was moderate, as the one included study had a low risk of bias in all but one domain.

#### Skills

A total of 24 studies addressed skill outcomes. Figure 5 summarizes the results of studies reporting skill outcomes.

#### Serious Gaming/Gamification Versus Traditional Learning

Twelve RCTs [21,22,27-31,35,37,38,42,45] and three cRCTs [19,23,41] compared serious gaming/gamification to traditional learning in this outcome category. The results were inconsistent, and studies were generally of low quality, making it difficult to draw conclusions about the efficacy of these interventions.

Six studies reported significant differences between groups for overall skill assessments in favor of serious gaming [27,28,35,37,42,45], with a magnitude of effect ranging from small to large. However, SMDs for two of these studies could not be calculated due to missing data [27,45]. Interventions included games with scenarios simulating clinical environments [27,28,35,37] and serious diverting interventions for improving practical skills [42].

Three studies comparing serious gaming/gamification with traditional learning used multiple measures for assessing skill outcomes; differences in favor of serious gaming/gamification were observed for some, but not all, of these skill measures, and the studies did not present an overall estimate of the effect [22,30,38]. Effect sizes could not be estimated, as SDs were not reported and attempts to contact the authors for further data were unsuccessful.

Two studies reported no significant difference in skill outcomes when comparing serious gaming/gamification and traditional learning and another reported no differences in pre- and posttest scores in either group [31].

Three cRCTs were also included [19,23,41]. One showed evidence of an effect of small magnitude, favoring a blood transfusion game group [41] (SMD: 0.33, 95% CI: –1.19 to 1.86); the second study found evidence of a moderate magnitude of effect, favoring the standard educational activity group, although skill measures were self-perceived as opposed to objective [19] (SMD: –0.77, 95% CI: –2.53 to 1.00); and the third showed no evidence of effect for a triage trainer game [23] (SMD: –0.18, 95% CI: –2.37 to 2.02). Each of these results may not be statistically significant.

There is some evidence that serious gaming/gamification interventions are more effective for improving skills than traditional learning. The quality rating assessed using GRADE was low, as the risk of bias was unclear for multiple domains and all the included studies had fewer than 400 participants.

#### Serious Gaming/Gamification Versus Other Modalities Of Digital Education

Five studies comparing skill outcomes for serious gaming/gamification and other modalities of digital education found no evidence of a difference in outcomes between groups [16,17,22,31,32]. In these studies, serious gaming was compared with an online video on dentin bonding [32] and with an electronic module (e-module) on patient safety [17] and management of an acutely unwell patient [16]. Another study reported higher postintervention skill score in a virtual reality control group than a commercial off-the-shelf intervention, with a large magnitude of effect for the time taken to complete surgical skill tasks (peg transfer and bimanual carrying; SMD: –1.56, 95% CI: –0.31 to –2.81), but reported no difference for distance travelled with surgical instruments when completing these tasks [14]. The quality rating assessed using GRADE was low.

#### Serious Gaming/Gamification Versus Serious Gaming/Gamification

We are uncertain whether any particular type of serious gaming/gamification is more effective than the other for improving skills. In three of the five studies comparing two serious gaming/gamification interventions, games involving motor skills, visuospatial skills, and manual dexterity may be more effective than interventions involving cognitive skills for improving laparoscopic surgical skills [24-26,33,34], but the quality of available evidence is very low.

### Professional Attitudes

#### Summary

**Figure 6:**
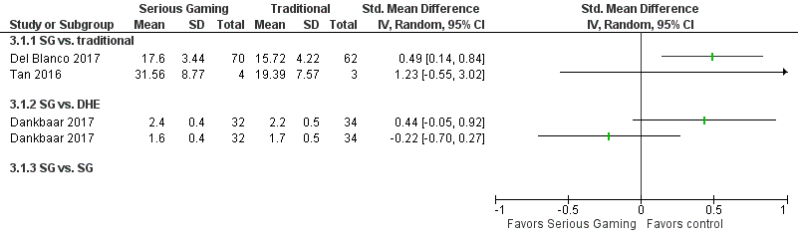
Forest plot for attitudes outcomes. IV: inverse variance; SG: serious games; DHE: digital health education.

Figure 6 summarizes the results of studies including professional attitudes outcomes.

Two RCTs compared a serious gaming/gamification intervention with traditional learning and measured outcomes related to professional attitudes. There was some evidence of a small magnitude of effect for a serious game, preparing students to go into the operating theatre for the first time, compared with traditional learning (SMD: 0.49, 95% CI: 0.14-0.84) [28]. A study comparing an insulin-prescribing game with an onsite learning activity for primary care reported insufficient data for comparisons between groups [43].

One cRCT was also included [41]. When reanalyzed with the number of clusters as the sample size to account for clustering in the analysis, there was evidence of intervention effectiveness, but this may not have been statistically significant and the analysis was likely underpowered (SMD: 1.23, 95% CI: –0.55 to 3.02). The quality of evidence for this outcome and comparison was rated very low according to the GRADE assessment.

#### Serious Gaming/Gamification Versus Other Modalities Of Digital Education

One study compared a serious game and an e-module on patient safety and reported no difference between groups in perceived patient safety behavior or reported stress [17].

The quality of evidence was rated low according to the GRADE assessment.

#### Serious Gaming/Gamification Versus Serious Gaming/Gamification

One study (reported in two papers) compared two serious diverting interventions, one was a first-person shooter (FPS) and one was a non-FPS, and reported no significant differences in self-efficacy or positive engagement modes [24,25]. Data were insufficient for calculation of effect sizes. The quality of the evidence was very low according to GRADE assessment.

### Satisfaction

#### Summary

Eleven RCTs [15,16,20-22,26,27,32,33,39,45] and two cRCTs measured outcomes relating to satisfaction [19,41]. Seven studies did not measure satisfaction in a comparison group [19,21,22,27,32,35,45], Diehl et al measured satisfaction with the intervention and comparison group using different scales [43], and Kerfoot et al reported results for participants in both groups combined [15]; therefore, these studies did not meet the inclusion criteria for this review. The remaining studies showed mixed evidence and are compared below.

#### Serious Gaming/Gamification Versus Traditional Learning

One study reported significantly better attitudes toward learning among a serious gaming group (a urology adventure game group) compared with a written script [20]. The quality rating assessed using GRADE was low.

#### Serious Gaming/Gamification Versus Other Modalities of Digital Education

Three studies reported higher satisfaction scores for serious gaming/gamification on managing acutely unwell patients [16], patient safety [17], and training during a pediatric clerkship [39] compared with an e-module [16,17] or Web-based flashcards [39] covering the same topics. The quality rating assessed using GRADE was low.

#### Serious Gaming/Gamification Versus Serious Gaming/Gamification

Results of a participant survey [26] suggested that more participants in the FPS gaming group than in the non-FPS gaming group found the intervention beneficial for their performance on a surgical simulator. No significance test was reported. The quality rating assessed using GRADE was very low.

### Secondary Outcomes

No studies measured economic outcomes of education or adverse effects of the intervention.

## Discussion

### Overview

The objective of this systematic review was to evaluate the effectiveness of serious gaming and gamification interventions for delivering pre- and postregistration health professions education. A total of 30 studies, most at high risk of bias according to Higgins [12], were identified, with high levels of heterogeneity in terms of populations and outcomes.

Serious gaming/gamification has the potential to reach a global audience and hence has been identified as a possible educational strategy that could contribute to transformation of health professions education. Results from our review show that serious gaming/gamification in pre- and post- registration health professions education could result in increased knowledge, skills, and satisfaction when compared to traditional education and, perhaps, other modalities of digital education.

Most of the current literature on the effectiveness of serious gaming/gamification has been performed in high-income countries, which limits the applicability of this review’s findings to low- and middle-income countries. This is a key gap in the evidence, as low-and middle-income countries are most affected by the worldwide shortage of trained health workers [46]. Other limitations of the evidence base include the lack of studies assessing patient outcomes, or clinician behavior.

The cost of serious gaming devices might be a barrier for use compared with traditional lectures or text books. For example, some of the included studies used game consoles, which many health care workers, particularly in low- and middle-income settings, may not have access to. Other included studies used lower-cost modes of delivery, such as projecting a serious game to a group of students who played together. However, none of the eligible studies provided any information about economic outcomes of education or adverse or unintended effects of the intervention, which limits our understanding of the feasibility of implementing these interventions in practice and our understanding of the applicability of serious gaming/gamification as a cost-effective solution.

Considering the types of interventions that may be effective, based on classification of interventions by original design intention, there were no clear patterns suggesting differing effectiveness between custom designed games and commercial off-the-shelf games for skill outcomes. Only custom-designed interventions were used to improve knowledge.

There was considerable heterogeneity in the results, particularly for skill outcomes, which we were unable to explain by systematic consideration of the types of intervention, population, and comparison group.

As serious gaming/gamification is an emerging field in the education sector, there are few previous reviews of the literature on its role in health professions education. Wang et al [47] conducted a systematic review of serious games for training health care professionals focused on game development and evaluation methodologies and reported a growing number of interventions and diversity of game genres over time [47]. Similar to our review, they found that study designs and methodological quality were heterogeneous and that best practices for development, evaluation, and use of such interventions are still being defined. A scoping review of serious gaming/gamification in health professions highlighted the need for economic evaluation of interventions, particularly when studies show no difference in efficacy between a serious game and traditional learning [48]. Our review contributes to the literature by providing an up-to-date summary of the evidence, focused on intervention effectiveness with a comprehensive systematic search. This is the first systematic review of the evidence indicating that serious gaming/gamification may improve participant knowledge compared with traditional learning.

There is a broad range of literature on serious gaming beyond health professions education. Meta-analyses have suggested that these interventions could significantly enhance learning among school students [49], adult workforce trainees [50], and mixed-age groups with regard to cognitive and attitudinal outcomes [51,52] and knowledge acquisition [53]. These reviews also suggested that games were more effective if they were supplemented with other methods of instruction, had multiple sessions, and involved active rather than passive learning. It was unclear whether playing as a group or alone was more effective. Systematic reviews have also suggested that serious gaming may have a role in the management of various medical conditions such as depression [54] and chronic conditions in young people [55] and in improving health outcomes [56]. The body of evidence on gamification interventions for education is smaller, with a systematic mapping study suggesting that most studies focused on the role of such interventions in student engagement and were published only as conference papers rather than full peer-reviewed articles [57]. A systematic review identified some evidence that gamification can be beneficial for health behavior change and well-being [58].

This review suggests that serious gaming may have the potential to advance education by improving knowledge, and possibly skill, outcomes for health professions compared with traditional learning. It may be able to provide educational interventions that are of equivalent educational value to other kinds of digital education, but with improved learner satisfaction. If this approach is equivalent to other kinds of education in terms of outcome but more cost-effective or able to offer greater access, it may provide further reasons to recommend serious gaming/gamification interventions, but no studies assessing these factors were identified.

Only two studies assessed gamification interventions. One suggested that the intervention was more effective than an online posting in improving knowledge by a large magnitude. The other suggested greater improvements in patient outcomes for questions spaced with four questions every 4 days rather than two questions every 2 days. These findings suggest that it may be worthwhile to incorporate gamification techniques into education, where possible, particularly for interventions aimed at improving knowledge, although further evidence is needed to establish the effectiveness among different groups of health professions for a wider range of patient outcomes and skill- and attitudes-related outcomes.

### Strengths and Limitations

This review adopted a detailed and comprehensive search strategy without language limitations, followed by robust screening, data extraction, and risk-of-bias assessments, adhering to the Cochrane guidelines [12]. Thirty studies were found to be eligible, but most of them were at high risk of bias according to Higgins [12], with high levels of heterogeneity in terms of populations and outcomes. This heterogeneity of the included studies made it inappropriate to perform meta-analysis for any outcomes. Evidence for the majority of the outcomes and comparisons in the review was considered of low quality. Many studies have small sample sizes that were unlikely to provide sufficient power to detect an effect, provided insufficient detail for complete risk of bias assessment, and did not report all data for all outcomes assessed; in addition, statistical analysis was often not performed appropriately for the data (eg, not accounting for clustering), reducing confidence in the results (Figures 2 and 3). Only two studies of gamification interventions were identified.

### Future Research

Serious gaming has the potential to contribute to the field of health professions education, but given that most studies to date are of low quality and carried out in high-income countries, future research should seek to use an RCT or cRCT design following a published protocol; evaluate interventions with a robust theoretical underpinning; be adequately powered; involve participants from low- and middle-income countries; appropriately randomize participants and blind outcome assessors, where possible; use validated outcome-assessment tools, facilitating comparability between interventions and studies; compare both serious gaming and gamification interventions with each other and with controls (other types of digital health education or traditional learning); and assess patient outcomes, participant behavior, attitudes, economic outcomes of education, and adverse events.

### Conclusions

There is some evidence that serious gaming/gamification may improve health professionals’ knowledge after the intervention compared with traditional education. In addition, some low-quality evidence shows that serious gaming/gamification may improve or be equivalent to traditional education for skills and to other modalities of digital education for knowledge and skills. Future research should evaluate theory-grounded interventions and assess patient outcomes, economic outcomes of education, and adverse events.

## Appendix

Multimedia Appendix 1 MEDLINE (Ovid) search strategy.

Multimedia Appendix 2 Outcome and results of included studies comparing serious gaming.

Multimedia Appendix 3 Outcome and results of included studies comparing serious gaming/gamification and other digital education approaches.

Multimedia Appendix 4 Outcome and results of included studies comparing serious gaming/gamification and another type of serious gaming/gamification intervention.

## Abbreviations

COTS: commercial off the shelf
cRCT: cluster randomized controlled trials
e-learning: electronic learning
e-module: electronic module
FPS: first-person shooter
OSCE: objective structured clinical examination
RCT: randomized controlled trials
SMD: standardized mean differences

## References

[1] Alvarez J, Djaouti D (2012). Serious Games: An Introduction.

[2] Alvarez J (2015). From Videogame to Serious Game: the concept of Serious diverting and Serious Modding.

[3] Wortley, David (2013). SlideShare.

[4] Deterding S, Dixon D, Khaled R, Nacke L (2011). Gamification: toward a definition.

[5] Akl EA, Kairouz VF, Sackett KM, Erdley WS, Mustafa RA, Fiander M, Gabriel C, Schünemann H (2013). Educational games for health professionals. Cochrane Database Syst Rev.

[6] Susi T, Johannesson M, Backlund P (2007). Serious Games - An Overview.

[7] Prensky M (2003). Digital game-based learning. Comput Entertain.

[8] Garris R, Ahlers R, Driskell J.E (2016). Games, Motivation, and Learning: A Research and Practice Model. Simulation & Gaming.

[9] Ruiz JG, Mintzer MJ, Issenberg SB (2006). Learning objects in medical education. Med Teach.

[10] Allery LA (2004). Educational games and structured experiences. Med Teach.

[11] Vogel M, Wood DF (2002). Love it or hate it? Medical students' attitudes to computer-assisted learning. Med Educ.

[12] Higgins J, Green S (2011). Cochrane Handbook for Systematic Reviews of Interventions. Version 5.1.0.

[13] Car J, Carlstedt-Duke J, Tudor Car L, Posadzki P, Whiting P, Zary N, Atun R, Majeed A, Campbell J, Digital Health Education Collaboration (2019). Digital Education in Health Professions: The Need for Overarching Evidence Synthesis. J Med Internet Res.

[14] Chien JH, Suh IH, Park S, Mukherjee M, Oleynikov D, Siu K (2013). Enhancing fundamental robot-assisted surgical proficiency by using a portable virtual simulator. Surg Innov.

[15] Kerfoot BP, Baker H (2012). An online spaced-education game for global continuing medical education: a randomized trial. Ann Surg.

[16] Dankbaar MEW, Alsma J, Jansen EEH, van Merrienboer JJG, van Saase JLCM, Schuit SCE (2016). An experimental study on the effects of a simulation game on students' clinical cognitive skills and motivation. Adv Health Sci Educ Theory Pract.

[17] Dankbaar MEW, Richters O, Kalkman CJ, Prins G, Ten Cate OTJ, van Merrienboer JJG, Schuit SCE (2017). Comparative effectiveness of a serious game and an e-module to support patient safety knowledge and awareness. BMC Med Educ.

[18] Graafland M, Bemelman WA, Schijven MP (2017). Game-based training improves the surgeon's situational awareness in the operation room: a randomized controlled trial. Surg Endosc.

[19] Lagro J, van de Pol MHJ, Laan A, Huijbregts-Verheyden FJ, Fluit LCR, Olde Rikkert MGM (2014). A randomized controlled trial on teaching geriatric medical decision making and cost consciousness with the serious game GeriatriX. J Am Med Dir Assoc.

[20] Boeker M, Andel P, Vach W, Frankenschmidt A (2013). Game-based e-learning is more effective than a conventional instructional method: a randomized controlled trial with third-year medical students. PLoS One.

[21] Hannig A, Lemos M, Spreckelsen C, Ohnesorge-Radtke U, Rafai N (2013). Skills-O-Mat: Computer Supported Interactive Motion- and Game-Based Training in Mixing Alginate in Dental Education. Journal of Educational Computing Research.

[22] Cook NF, McAloon T, O'Neill P, Beggs R (2012). Impact of a web based interactive simulation game (PULSE) on nursing students' experience and performance in life support training--a pilot study. Nurse Educ Today.

[23] Knight JF, Carley S, Tregunna B, Jarvis S, Smithies R, de Freitas S, Dunwell I, Mackway-Jones K (2010). Serious gaming technology in major incident triage training: a pragmatic controlled trial. Resuscitation.

[24] Hedman L, Schlickum M, Felländer-Tsai L (2013). Surgical novices randomized to train in two video games become more motivated during training in MIST-VR and GI Mentor II than students with no video game training. Stud Health Technol Inform.

[25] Schlickum MK, Hedman L, Enochsson L, Kjellin A, Felländer-Tsai L (2009). Systematic video game training in surgical novices improves performance in virtual reality endoscopic surgical simulators: a prospective randomized study. World J Surg.

[26] Kolga SM, Hedman L, Enochsson L, Kjellin A, Felländer-Tsai L (2008). Transfer of systematic computer game training in surgical novices on performance in virtual reality image guided surgical simulators. Stud Health Technol Inform.

[27] Boada I, Rodriguez-Benitez A, Garcia-Gonzalez JM, Olivet J, Carreras V, Sbert M (2015). Using a serious game to complement CPR instruction in a nurse faculty. Comput Methods Programs Biomed.

[28] Del Blanco A, Torrente J, Fernández-Manjón B, Ruiz P, Giner M (2017). Using a videogame to facilitate nursing and medical students' first visit to the operating theatre. A randomized controlled trial. Nurse Educ Today.

[29] Foss B, Lokken A, Leland A, Stordalen J, Mordt P, Oftedal BJ (2014). Digital Game-Based Learning: a supplement for medication calculation drills in nurse education. E-Learning and Digital Media.

[30] Giannotti D, Patrizi G, Di Rocco G, Vestri AR, Semproni CP, Fiengo L, Pontone S, Palazzini G, Redler A (2013). Play to become a surgeon: impact of Nintendo Wii training on laparoscopic skills. PLoS One.

[31] Adams BJ, Margaron F, Kaplan BJ (2012). Comparing video games and laparoscopic simulators in the development of laparoscopic skills in surgical residents. J Surg Educ.

[32] Amer RS, Denehy GE, Cobb DS, Dawson DV, Cunningham-Ford MA, Bergeron C (2011). Development and evaluation of an interactive dental video game to teach dentin bonding. J Dent Educ.

[33] Ju R, Chang PL, Buckley AP, Wang KC (2012). Comparison of Nintendo Wii and PlayStation2 for enhancing laparoscopic skills. JSLS.

[34] Ju R, Chang PL, Buckley AP, Wang KV (2011). Is Nintendo Wii a More Suitable Video Game Platform Than Playstation 2 for Enhancing Laparoscopic Skills?. Journal of Minimally Invasive Gynecology.

[35] Katz D, Zerillo J, Kim S, Hill B, Wang R, Goldberg A, DeMaria S (2017). Serious gaming for orthotopic liver transplant anesthesiology: A randomized control trial. Liver Transpl.

[36] Kerfoot BP, Turchin A, Breydo E, Gagnon D, Conlin PR (2014). An online spaced-education game among clinicians improves their patients' time to blood pressure control: a randomized controlled trial. Circ Cardiovasc Qual Outcomes.

[37] LeFlore J, Anderson M, Zielke MA, Nelson KA, Thomas PE, Hardee G, John LD (2012). Can a virtual patient trainer teach student nurses how to save lives--teaching nursing students about pediatric respiratory diseases. Simul Healthc.

[38] Plerhoples TA, Zak Y, Hernandez-Boussard T, Lau J (2011). Another use of the mobile device: warm-up for laparoscopic surgery. J Surg Res.

[39] Sward KA, Richardson S, Kendrick J, Maloney C (2008). Use of a Web-based game to teach pediatric content to medical students. Ambul Pediatr.

[40] Gauthier A, Corrin M, Jenkinson J (2015). Exploring the influence of game design on learning and voluntary use in an online vascular anatomy study aid. Computers & Education.

[41] Tan AJQ, Lee CCS, Lin PY, Cooper S, Lau LST, Chua WL, Liaw SY (2017). Designing and evaluating the effectiveness of a serious game for safe administration of blood transfusion: A randomized controlled trial. Nurse Educ Today.

[42] de Araujo TB, Silveira FR, Souza DLS, Strey YTM, Flores CD, Webster RS (2016). Impact of video game genre on surgical skills development: a feasibility study. J Surg Res.

[43] Diehl LA, Souza RM, Gordan PA, Esteves RZ, Coelho ICM (2017). InsuOnline, an Electronic Game for Medical Education on Insulin Therapy: A Randomized Controlled Trial With Primary Care Physicians. J Med Internet Res.

[44] Rondon S, Sassi FC, Furquim de Andrade CR (2013). Computer game-based and traditional learning method: a comparison regarding students' knowledge retention. BMC Med Educ.

[45] Li J, Xu Y, Xu Y, Yue P, Sun L, Guo M, Xiao S, Ding S, Cui Y, Li S, Yang Q, Chang P, Wu Y (2015). 3D CPR Game Can Improve CPR Skill Retention. Stud Health Technol Inform.

[46] (2018). World Health Organization.

[47] Wang R, DeMaria S, Goldberg A, Katz D (2016). A Systematic Review of Serious Games in Training Health Care Professionals. Simul Healthc.

[48] Ricciardi F, De Paolis LT (2014). A Comprehensive Review of Serious Games in Health Professions. International Journal of Computer Games Technology.

[49] Clark D, Tanner-Smith E, Killingsworth S (2016). Digital Games, Design, and Learning: A Systematic Review and Meta-Analysis. Rev Educ Res.

[50] Sitzmann T (2011). A meta-analytic examination of the instructional effectiveness of computer-based simulation games. Personnel Psychology.

[51] Vogel J, Vogel D, Cannon-Bowers J, Bowers C, Muse K, Wright M (2016). Computer Gaming and Interactive Simulations for Learning: A Meta-Analysis. Journal of Educational Computing Research.

[52] Wouters P, van Nimwegen C, van Oostendorp H, van der Spek Ed (2013). A meta-analysis of the cognitive and motivational effects of serious games. Journal of Educational Psychology.

[53] Boyle E, Hainey T, Connolly T, Gray G, Earp J, Ott M, Lim T, Ninaus M, Ribeiro C, Pereira J (2016). An update to the systematic literature review of empirical evidence of the impacts and outcomes of computer games and serious games. Computers & Education.

[54] Fleming T, Cheek C, Merry S, Thabrew H, Bridgman H, Stasiak K, Shepherd M, Parry Y, Hetrick S (2014). Serious games for the treatment or prevention of depression: a systematic review. Revista de Psicopatologia y Psicologia Clinica.

[55] Charlier N, Zupancic N, Fieuws S, Denhaerynck K, Zaman B, Moons P (2016). Serious games for improving knowledge and self-management in young people with chronic conditions: a systematic review and meta-analysis. J Am Med Inform Assoc.

[56] Primack B, Carroll M, McNamara M, Klem M, King B, Rich M, Chan C, Nayak S (2012). Role of video games in improving health-related outcomes: a systematic review. Am J Prev Med.

[57] De Sousa Borges S, Durelli V, Reis H, Isotani S (2014). A systematic mapping on gamification applied to education.

[58] Johnson D, Deterding S, Kuhn K, Staneva A, Stoyanov S, Hides L (2016). Gamification for health and wellbeing: A systematic review of the literature. Internet Interv.

