# Supplementary material for: Serious Gaming and Gamification Education in Health Professions: Systematic Review
Source: J Med Internet Res. 2019 Mar 28;21(3):e12994. doi: 10.2196/12994 (PMC6458534; doi:10.2196/12994)
Supplement: Multimedia Appendix 2 [file jmir_v21i3e12994_app2.pdf]

Multimedia Appendix 2 Outcome and results of included studies comparing serious gaming/gamification with traditional learning ***(see key below table for acronyms)***

**Study ID**	**Outcome (measurement tool)**	**Results (SMD calculated where sufficient data reported or p values, [95% confidence interval]**
**Adams 2012**	Skills (peg transfer time)	No significant difference between pre- and post-test scores in any group
**Boada 2015**	Skills (CRP lab assessment)	Those in the intervention group who completed >50% of tasks vs. control p<0.05
**Boeker 2013**	Knowledge (34 question single choice test)	SMD 0.69 [0.35, 1.03]
	Satisfaction (Likert scale)	Serious gaming vs. traditional learning p<0.001
**Cook 2012**	Skills (assessment of ABCDE approach)	Significant differences between groups for three of eight measures
**De Araujo 2016**	Skills (standardized surgical skills exercise)	SMD 2.07 [0.83, 3.31]
**Del Blanco 2017**	Knowledge (participant perceived knowledge measured with a Likert scale)	SMD 1.05 [0.68, 1.41]
	Skills (perceived errors)	SMD 0.38, 95% CI 0.04 to 0.73
	Attitudes (fear to make mistakes and attitudes to patients/staff measured with Likert scales)	SMD 0.49 [0.14, 0.84]

Study ID	Outcome (measurement tool)	Results (SMD calculated where sufficient data reported or p values, [95% confidence interval]
**Diehl 2017**	Knowledge (MCQs)	SMD 0.40 [0.06, 0.73]
	Attitudes (Likert scale)	Attitudes improved significantly in 4 out of 9 items in the intervention group and 3 out of 9 items in the control group
**Foss 2014**	Skills (medication calculation assessment)	Report no significant difference
**Giannotti 2013**	Skill (basic laparoscopic skills assessment on a validated simulator)	Statistically significant differences reported for a variety of tasks but not all and no overall measure
**Graafland 2017**	Skills (assessment using OSATS on procedures conducted on a live anaesthetized pig model)	Statistically significant difference in total problems solved (p=0.04)
**Hannig 2013**	Skills (self-report skills in alginate mixing, mean score for 6 item Likert scale)	SMD 0.08 [-0.45 to 0.61]
**Katz 2017**	Skills (graded on a simulation scenario)	SMD 0.76, 95% CI 0.11 to 1.40
	Satisfaction (game group only)	*
**Knight 2010**	Skills (time to triage in an outdoor gas explosion scenario assessment)	SMD -0.18 [-2.37 to 2.02]
**Lagro 2014**	Knowledge (self-perceived, Likert scale)	SMD 0.01 [-1.5, 1.61]

Study ID	Outcome (measurement tool)	Results (SMD calculated where sufficient data reported or p values, [95% confidence interval]
	Skills (self-perceived)	SMD -0.77 [-2.53, 1.00]
	Satisfaction (intervention only, Likert scale)	*
**LeFlore 2012**	Knowledge (MCQ)	SMD 0.65 [0.23, 1.07]
	Skills (OSCE)	SMD 0.92 [0.49 to 1.35]
**Li 2015**	Skills (mannequin based assessment)	Serious gaming vs. control $p<0.05$
**Plerhoples 2011**	Skills (laparoscopic simulation)	Statistically significant differences reported for a variety of tasks but not all and no overall measure
**Rondon 2013**	Knowledge (MCQs)	SMD 0.05 [-0.74, 0.83]
**Tan 2016**	Knowledge (MCQs)	SMD 1.95 [-0.20 to 4.11]
	Skills (performance tool)	SMD 0.33, 95% [CI -1.19 to 1.86]
	Attitudes (blood transfusion confidence scale)	SMD 1.23 [-0.55, 3.02]

OSATS = Objective structured assessment of technical skills

OSCE = Objective Structured Clinical Examination

MCQ = Multiple Choice Question

SMD = Standardized mean difference
