# Supplementary material for: Serious Gaming and Gamification Education in Health Professions: Systematic Review
Source: J Med Internet Res. 2019 Mar 28;21(3):e12994. doi: 10.2196/12994 (PMC6458534; doi:10.2196/12994)
Supplement: Multimedia Appendix 3 [file jmir_v21i3e12994_app3.pdf]

Multimedia Appendix 3 Outcome and results of included studies comparing serious gaming/gamification with other digital education interventions ***(see key below table for acronyms)***

Study ID	Outcome (measurement tool)	Results (SMD calculated where sufficient data reported or p values, [95% confidence interval]
**Amer 2011**	Knowledge (procedural knowledge of resin bonding)	SMD -0.22 [-0.66 to 0.23]
	Skills (dentin shear bond strength)	SMD 0.00 [-0.45, 0.44]
**Chien 2013**	Skills (time taken for peg transfer and bi-manual carrying tasks)	SMD -1.56 [-0.31, -12.81]
	Skills (distance travelled for peg transfer and bi-manual carrying tasks)	SMD 0.96 [-0.17, 2.09]
**Dankbaar 2016**	Skills (mannequin based scenario global assessment)	SMD -0.15 [-0.66, 0.36]
	Satisfaction (engagement and experience of feedback)	P<0.001<br>P<0.05
**Dankbaar 2017**	Knowledge (70 MCQs)	SMD 0.33 [-0.16 to 0.82]
	Skills (self-efficacy for recognition and communication of patient safety issues)	No significant difference between groups
	Attitudes (perceived patient safety behavior)	SMD 0.44 [-0.05, 0.92]
	Attitudes (perceived stress)	SMD -0.22 [-0.70, 0.27]

Study ID	Outcome (measurement tool)	Results (SMD calculated where sufficient data reported or p values, [95% confidence interval]
	Satisfaction (intervention evaluation questionnaire)	SG rated significantly better for engagement (p<0.001) and ease of use (p<0.01) but not 'educational'
**Gauthier 2015**	Knowledge (vascular anatomy knowledge test)	SMD 0.24 [-0.34 to 0.82]
**Kerfoot 2014**	Patient outcomes (time to blood pressure control extracted from regional database)	p=0.46<br>(p=0.022 for subgroup already on BP medication)
	Knowledge (24 clinical scenario based questions)	SMD 0.82 [0.42 to 2.11]
**Sward 2008**	Knowledge (100 item test)	SMD 0.14 [-0.31 to 0.59]
	Satisfaction (variety of measures)	Statistically significant difference for some measures but not all

SMD = Standardized mean difference

BP = Blood pressure
