# Supplementary material for: Serious Gaming and Gamification Education in Health Professions: Systematic Review
Source: J Med Internet Res. 2019 Mar 28;21(3):e12994. doi: 10.2196/12994 (PMC6458534; doi:10.2196/12994)
Supplement: Multimedia Appendix 4 [file jmir_v21i3e12994_app4.pdf]

Multimedia Appendix 4 Outcome and results of included studies comparing serious gaming/gamification with another type of serious gaming/gamification intervention *(see key below table for acronyms)*

**Study ID**	**Outcome (measurement tool)**	**Results (SMD calculated where sufficient data reported or p values, [95% confidence interval]**
**Adams 2012**	Skills (peg transfer time)	No significant difference between pre- and post-test scores in any group
**De Araujo 2016**	Skills (standardized surgical skills exercise)	SMD -0.86 [-2.19, 0.47] (Shot G vs. Surg G)
		SMD 0.72 [-0.59, 2.02] (Surg G vs. RaceG)
		SMD 1.70 [0.13, 3.27] (Shot G vs. Race G)
		SMD 2.07 [0.83, 3.31] (SG vs. traditional)
**Hedman 2013/ Kolga Schlickum 2009**	Skills (assessed on a surgical simulator)	FPS superior on a range of measures, but not all
	Attitudes (self-efficacy and positive engagement modes)	No significant differences
**Ju 2011/12**	Skills (bead transfer time during assessment on laparoscopic box simulator)	SMD 1.54 [0.84, 2.24]
	Skills (suturing score during assessment on laparoscopic box simulator)	SMD 0.21 [-0.40, 0.82]

**Kerfoot 2012**	Knowledge (percentage of game questions scored correctly)	SMD 0.50 [0.38, 0.64]
**Kolga Schlickum 2008**	Skills (assessment on a surgical simulator exercise)	FPS vs. non-FPS p=0.04
	Satisfaction	Participant survey reported greater satisfaction in the intervention group, but no significance test reported

FPS = First person shooter

SMD = Standardized mean difference
